# New HIV testing technologies in the context of a concentrated epidemic and evolving HIV prevention: qualitative research on HIV self-testing among men who have sex with men and transgender women in Yangon, Myanmar

**DOI:** 10.7448/IAS.20.01.21796

**Published:** 2017-04-25

**Authors:** Andrea L. Wirtz, Emily Clouse, Vanessa Veronese, Kaung Htet Thu, Soe Naing, Stefan D. Baral, Chris Beyrer

**Affiliations:** ^a^ Center for Public Health and Human Rights, Department of Epidemiology, Johns Hopkins School of Public Health, Baltimore, MD, USA; ^b^ Centre for Population Health, Burnet Institute, Melbourne, Australia; ^c^ Department of Epidemiology and Preventative Medicine, Monash University, Melbourne, Australia; ^d^ International HIV/AIDS Alliance in Myanmar, Yangon, Myanmar

**Keywords:** men who have sex with men, transgender women, HIV test, Myanmar

## Abstract

**Introduction**: Global effort to increase early diagnosis and engagement in HIV care emphasize the importance of developing novel approaches to reaching those missed by traditional methods. Such needs are particularly great for men who have sex with men (MSM), transgender women (TW), and other populations who face stigma. Myanmar’s HIV epidemic is concentrated among key populations and the revised National Strategy aims to reduce late diagnosis and barriers to care to curb HIV incidence among these groups. HIV self-testing (HIVST) may be one method to improve testing and diagnosis among key populations, by placing HIV testing and disclosure within the individual’s control.

**Methods**: Formative, qualitative research including in-depth interviews with adult MSM (N = 12) and TW (N = 13) and focus group discussions with MSM, TW, and community key informants (N = 35) were conducted in June-September 2015 in Yangon, Myanmar. To inform a subsequent HIV care continuum intervention, including HIVST, participants’ opinions and perceptions about HIVST were elicited.

**Results**: The confidentiality and privacy of HIVST, particularly as it related to disclosure of HIV status and sexual behaviour, was widely recognized among participants. These major advantages were further supported by the opportunity to avoid stigma, convenience of self-testing (reduced need for transportation and time to go to clinics), and the availability of a pain-free testing option. Participants weighed these benefits against perceived disadvantages of HIVST, the majority of which centred on the perception that HIVST does not include counselling. Participants were concerned that potential lack of counselling would result in poor mental health outcomes, inadequate linkage to HIV care and surveillance, and reductions in disclosure of HIV status. Participants did not view these disadvantages as an impediment, but provided suggestions for future implementation of HIVST in Myanmar.

**Conclusions**: MSM and TW are optimistic about the confidentiality and privacy afforded by HIVST but wanted HIV counselling and linkage to appropriate services. The domestic reprioritization of HIV and opening of the country to international support has substantially increased the availability of HIV treatment and provides new opportunities, like HIVST, to potentially improve the HIV response for key populations who are at risk for HIV acquisition.

## Introduction

Demonstration that early antiretroviral therapy (ART) can result in up to 96% reduction in HIV transmission [1] has led to a global goal to increase early identification and engagement in HIV care among people living with HIV. These efforts include the 90-90-90 strategy proposed by the Joint United Nations Programme on HIV/AIDS (UNAIDS) as well as many national strategies that target high coverage of diagnosis, treatment and viral suppression among people living with HIV in attempts to reduce HIV mortality and morbidity and ultimately end the HIV epidemic [2,3]. Several new prevention technologies, most notably pre-exposure prophylaxis (PrEP), are also HIV status dependent and rely on regular HIV testing for those at risk. With the focus on testing has come the need for innovative methods to reach and identify people who are at risk for HIV acquisition. These innovative methods are even more important for key populations affected by HIV, such as men who have sex with men (MSM) and transgender women (TW), who may be stigmatized on the basis of their sexuality or gender identity and dually stigmatized when found to be living with HIV. Stigma has served as a formidable barrier to HIV testing, prevention and care for MSM and TW and has resulted in lower access to or uptake of HIV prevention, testing, and care services and high levels of undiagnosed infection in many settings [4–9].

HIV self-testing (HIVST) has emerged as one such innovative method to identifying new cases or previously undiagnosed cases of HIV infection. HIVST may also serve an important role for HIV status-dependent interventions, including PrEP, by allowing those with a preliminary negative HIVST result to engage in services where eligibility for PrEP can be further assessed [10]. Salivary-based rapid HIV tests generally have sensitivity of 91.7% and specificity of 97.9% and have been approved by the United States (U.S.) Food and Drug Administration (FDA) for home use due to their potential to reduce undiagnosed HIV infections [11]. Rapid HIV tests allow individuals to conduct rapid screening tests easily and privately at the location of their choosing, obtain preliminary HIV test results in settings that are free from potential stigma and discrimination, support individual self-efficacy to care for their health, and reduce travel and waiting times associated with facility-based testing [10,12–17]. In contexts where sexual behaviours or HIV status may be stigmatized, self-testing provides a promising alternative to provider-implemented testing programs.

Research on the feasibility and acceptability of HIVST among MSM and TW has predominantly originated from Europe and the U.S., where self-testing has been approved and available over-the-counter since 2012 [11]. Some studies have recently been reported from the Latin American and sub-Saharan African regions, as well as China and Cambodia [18–20]. A review on the acceptability and feasibility of self-testing among key populations has found general acceptance of HIVST among MSM, particularly noting convenience and privacy of testing [18]. Further pilot studies have also demonstrated the potential impacts of self-testing on HIV status awareness as well on behavioural risks among MSM [21,22].

The HIV epidemic in Southeast Asia is primarily concentrated among key populations, including people who use drugs, gay men and other MSM, TW, and sex workers. With the exception of a few countries, such as Thailand, HIV research among key populations is only recently emerging and stigmatization of gender identity and sexual orientation remains high [23]. In Myanmar, HIV is highly prevalent among MSM and TW, with combined prevalence estimates for these two groups reported at 11.6% nationally and reaching 26.6% in Yangon in 2015, and is potentially exacerbated by stigmatization of both HIV and same sex practices [2,24,25]. Access to HIV services is generally low, with less than 50% of MSM and TW reporting any history of HIV testing, according to the National AIDS Programme, highlighting the potential for new approaches, such as HIVST, to improve HIV testing rates [26]. Domestic reprioritization of HIV programmes and opening of the country to international engagement has substantially increased the availability of HIV treatment and provided new opportunities to address the epidemic among MSM and TW. In this context, little is known about the effectiveness of innovative methods to confidentially engage these populations in HIV testing, care, and treatment. As part of a larger implementation science study, we sought to conduct formative, qualitative research among MSM and TW in Myanmar to investigate new approaches, such as HIVST, to successfully identify and engage MSM and TW in HIV testing, care, and treatment [27].

## Methods

Qualitative data collection was conducted between June and September 2015 among adult MSM, TW, and key informants in Yangon, Myanmar.

### Study site

The study was conducted in Yangon, Myanmar. Yangon, formerly the capital of Myanmar, remains an important urban centre and is the largest city in Myanmar with a population exceeding 7.3 million persons in 2014 [28]. There is growing recognition and acceptance of LGBT populations in Yangon and a substantial emergence of community-based HIV prevention and care services for MSM and TW in Yangon. Interviews and focus group discussions were conducted in private office spaces of affiliated NGOs serving key populations in Yangon. Offices were geographically dispersed across the Yangon metropolitan area, allowing participants living across various locations of the city to participate in the study.

### Study sample

Adult MSM and TW were recruited via purposive sampling to participate in in-depth interviews and focus group discussions. While there are emerging and increasingly open gay communities in Yangon, the traditional categories of sexual orientation and gender identity in Myanmar culture are blurred; locally, the term MSM is often used interchangeably across male and transgender-identified populations and these groups often attend similar community-based events [29]. Inclusion criteria for MSM and TW included: aged 18 years or older, assigned male at birth, reports anal sex with a man in the last 12 months, speaks Myanmar, and is a current resident of the greater Yangon Region.

Focus group discussions were held among key informants, including MSM and TW community leaders and service providers providing HIV prevention and care to MSM and TW. Those unwilling or unable to participate in a focus group discussion were invited to participate in an in-depth interview. Key informants were required to be 18 years of age or older, report serving the MSM and/or TW community for at least one year in the greater Yangon area, and speak Myanmar.

All participants were recruited by word of mouth from partner NGOs using maximum variation purposive sampling methods, with an aim to include a range of sexual and gender identities, ages, and HIV status. Data collection ended when information saturation was achieved, as determined by redundancy in the content of discussions. In total, 12 in-depth interviews were conducted among MSM, 13 among TW, and five focus group discussions with MSM, TW, and key informants (N = 35). Table 1 provides a description of participant characteristics.

**Table 1.** Characteristics of MSM, TW, and key informant participants of in-depth interviews and focus group discussions in Yangon, Myanmar

**In-depth interview participants**	***n***	**%**
Median age, years (range)	23	(18–42)
MSM	12	48
TW	13	52
Sex worker	2	8
Living with HIV infection	8	32
HIV uninfected	16	64
HIV status unknown	1	4
**Focus group participants (N)**	**Characteristics**	
MSM community leaders (N = 9)	MSM outreach worker, peer educator, peer educator supervisor, volunteer, community worker	
Hidden MSM (N = 6)	MSM, living with HIV and HIV uninfected, low and middle income	
Service providers (N = 8)	MSM outreach worker, peer educator, counsellor, peer educator supervisor	
Service providers (N = 6)	general practitioners, sexual reproductive health educator, peer educators, outreach worker	
Transgender women (N = 6)	TW, living with HIV and HIV uninfected, sex worker	

### Data collection

Interviewers were MSM or TW themselves and had previous experience in HIV research or providing HIV services (e.g. several served as peer navigators to support engagement in HIV treatment and care for MSM and TW). Interviewers and facilitators underwent extensive training on the purposes of the study and qualitative research; qualitative research methods and probing; data management and security; and confidentiality and human subjects protections.

Prior to data collection, interviewers/facilitators conducted a private consent process to describe the study purpose and procedures with candidate participants. Participants were then asked to acknowledge consent by providing their name, code name, or other mark on the consent form. All data collection was anonymous and recorded interviews were transferred daily to password protected computers and subsequently erased from recording devices to protect study data. Discussions and interviews lasted approximately 60–90 min and were conducted in Myanmar language. Participants were provided with 5000 kyat ($4 USD) to reimburse their time and travel associated with participation.

### Measures and analyses

Interviews followed semi-structured interview guides and included the following domains: individual characteristics; social context for MSM and TW, including experiences of stigma; availability, experiences, and types of HIV testing for MSM and TW; opinions and knowledge about HIVST; and preferences for HIV prevention and care services for MSM and TW. Focus group discussions among MSM, TW, and service providers included the same domains but attempted to avoid discussion of individual information, particularly sensitive personal experiences or information. Focus group guides collected information on the following domains: types of services provided by the individual/organization where they worked; general discussion of clientele characteristics; experiences, protocols, and types of services available for providing HIV testing, linkage to care, and treatment for MSM and TW living with HIV. During interviews and focus group discussions, participants were provided with a description of HIVST, including how the HIVST worked, that counselling would be provided when HIVST kits were distributed, a description of HIVST accuracy and that confirmatory testing would be required, and were presented with a self-testing kit and instructions to see how the test operated.

Interviews and discussions were recorded and subsequently transcribed by a certified transcription agency, and translated to English. English language transcripts were reviewed by Myanmar study staff (KHT) against the original transcripts to ensure accuracy. Anonymous English language audio transcriptions and summary notes were entered into Atlas.ti software (Cincom Systems, Berlin) for coding and analysis. Research team members (AW, EC, VV) collectively coded five interviews to assess consistency across team members. Once consistency was achieved each transcript was coded separately by study team members and then reviewed during coding meetings. Topical codes were applied to allow quotations to be sorted according to interview guide domains and open interpretive coding was utilized to identify and analyze any emerging themes observed within and between topical areas. To support interpretation of findings, member checking was performed by hosting a validation workshop in September 2016 in which results of the analyses were presented to community interviewers/facilitators and other members of partner NGOs serving MSM and TW.

Findings present perceptions, potential acceptability, and opinions of HIV self-testing among MSM and TW. Quotations provided in the Tables and Supplementary Files (referenced below as S#) were selected to highlight themes emerging from the analysis; some quotations provide information about one theme in particular, while others may provide information relevant to several themes.

### Human subjects protections

This study was a partnership between Johns Hopkins University School of Public Health and the International HIV/AIDS Alliance in Myanmar, which provides HIV prevention and care services across Yangon and other regions in Myanmar, and three community-based organizations: Lotus organization, Phoenix Association and *Aye Nyein Myitta* that provide services to all people living with HIV (PLHIV) including MSM and TW in Yangon. The study was approved by the Johns Hopkins School of Public Health Institutional Review Board and the Myanmar Department of Medical Research, Ethical Review Committee.

## Results

Most participants generally believed that HIVST would be beneficial to MSM and TW in Yangon. However, HIVST was not universally acceptable among MSM, TW, and key informants. Rather, discussions around HIVST stimulated feedback about both the potential advantages and disadvantages of HIVST for MSM and TW in Myanmar and provided information that is informative for planning self-testing programs.

### Perceived advantages of self-testing

The most salient perception reported among interviews and discussions MSM, TW, and key informants were the apparent benefits and the privacy and confidentiality of HIVST (Table 2).

“I can do it just by myself. No matter what the result is, I will be the only one who knows it. No one else will know it.” IDI 19, MSM, age 18

**Table 2.** Perceived advantages of HIV self-testing: key findings and supporting quotations from MSM, TW, and key informants in Yangon, Myanmar

Topic	Relevant quotation
Confidentiality and privacy	*“I’m just saying as this is a discussion about MSM… the majority are afraid to go to a clinic to get the test. They are afraid that others might see them – because you can never be alone in a clinic where you get the test. Most of the times, there are a lot of clients/people there. As they are going to do the test at home, it can be better. Yes.”* *–FGD 2, Participant 5, hidden MSM*
Individual control over disclosure of sexual identity and/or HIV status	*“[Interviewer – Why do you want to use it [self-test]?] “What do I say? I don’t want my neighbors/community to know. I don’t want my family know either. So, this device is really confidential/secure for me. That’ why I want to use it.”* *– IDI37, TW, age 28, living with HIV*
Individual control over timing, location, and presence of others while testing	*“For those who are too shy to go and get a blood test, that new method can work. Let’s say a client goes and gets the test as a hidden MSM. As it is an HIV test, he would be worried that people might be watching him. For this kind of people, it is good to do the test at home – because he would do the test. And the result well most MSM there are many MSM who study about this knowledge. So, he can do self-testing at home. And he can also have good ideas/options on how to proceed after that. So, they do it at home. And he alone will know the result. And the organization he contacts will know it as well.”* – *FGD 2, Participant 5, hidden MSM*
Reduced time associated with facility-based testing	*“(existing test methods are) time consuming and there are all kinds of people there at the time of testing. I go there alone but I have to wait because there are other people coming for tests. It’s a little uncomforTable This new test will be great.”* *– IDI21, TW, age 31*
Pain-free testing method	*“If it [self-testing] is confidential/secure, not only me, everyone will get the test. As there is full confidentiality, nobody will know about us. And, as you test the saliva, there is less physical pain. Other test method tests blood and the needle prick is painful … to some MSMs….”* *– IDI16, MSM, age 23*

Among MSM and TW, the importance of privacy was particularly relevant in preventing family, neighbours, and other MSM and TW from finding out about one’s sexuality or HIV status. The confidentiality of self-testing was weighed against the uncertainty and concern faced by MSM about who may be present when seeking facility-based HIV testing and the potential for stigmatization (Supplementary Files, Quote S1).

The ease of testing in a time and location that is convenient to MSM and TW were additional benefits associated with HIVST. Moreover, the availability of a pain-free oral method eased fears associated with needles and finger-prick methods (Supplementary Files, Quote S2).

### Perceived disadvantages of HIV self-testing

Concerns were raised about the potential drawbacks of HIVST, including among those participants who also described potential benefits of this approach (Table 3). Most universally was the concern for poor mental health outcomes among MSM and TW who may learn about their HIV infection through self-testing.

If he has knowledge, he can struggle to stand on his feet. He doesn’t need to disclose his family. He can take treatment. If it gets into the hands of a person who doesn’t have knowledge, he might do something like suicide. I think pros and cons go along. FGD 5, Participant 1, TW

**Table 3.** Perceived disadvantages of HIV self-testing: key findings and supporting quotations from MSM, TW, and key informants in Yangon, Myanmar

Topic	Relevant quotation
Poor mental health outcomes	*“Students start having sex from 17–18. What will you do if such device is available and they try it? What will you do if they have it? They won’t know what to do. Some will be like it won’t get any better and they will keep doing it. What if they commit suicide? I don’t think it should go outside. It should be kept in the hands of doctors (under the care of doctors).”* *– FGD5, Participant 2, TW*
Potential deficiency of counseling and linkage to HIV care	*“Now there are many organizations for blood tests and medicines without charges. Even though these are free, majority don’t come to collect medicines. Some feel ashamed, some others are scared and others feel inferior… And it is also because people look down on you. Therefore, even when these NGOs are doing a lot by checking with doctors and providing counseling, they [MSM] don’t come to collect their medicines. If one does self-testing of his saliva, it will not work.”* *– IDI 26, TW, age 23*
Impaired surveillance and undisclosed HIV infection	*“I will talk about the disadvantages first. If they are going to test with it easily, data will be reduced. The number of HIV infections in Myanmar will be reduced. They [users] will not tell the truth… They will buy it and test it by themselves. If they don’t have it, they might tell. If they have it, they will not tell. Since they don’t tell the truth, data collection will be weak. Some will rather let themselves die. They will go somewhere no one know and let themselves die. Some will have sex intentionally unprotected.”* *–IDI 27 MSM, age 24*
Incorrect use will result in an inaccurate result	*“It can be a little difficult as I have to do it myself. But to get the result in 20 minutes would depend on how I do it. If there is any mistake, I will get a wrong result.”* *– IDI 19, MSM, age 18*
Misunderstanding that saliva does transmit HIV infection, if an oral swab can be used to test for HIV	*“Today, what we know is that HIV is not transmitted through saliva or kissing. But if we say testing the saliva can transmit the virus people might get worried that they might get infected through some saliva droplets while speaking. I am worried that that kind of message will spread.”* *– FGD 3, participant 3, MSM community service providers*

This concern was often tied to the perception that self-testing was conducted in absence of any counselling, instruction, or linkage to care (Supplementary Files, Quote S3). Despite these concerns, a few MSM and TW felt that the topic of HIV and the benefits of treatment were well known and would not result in suicidal intention or poor mental health outcomes (Supplementary Files, Quote S4).

Several participants perceived that counselling would not be provided with HIVST and felt that this lack of counselling would result in non-disclosure of one’s HIV status, impair HIV surveillance, and reduce access to HIV care. Few other MSM, TW, and key informants voiced concern that without appropriate counselling and linkage to care, some MSM and TW may knowingly or intentionally have unprotected sex after learning of their HIV infection (Supplementary File, Quote S4). Facility-based testing, on the other hand, particularly those provided by NGOs was known to provide MSM and TW with access to ART and other HIV-related care (Supplementary File, Quotes S5-7).

Beyond psychosocial impacts of HIVST, logistical concerns and accuracy of HIVST methods were a concern. In this case, some participants reported fear and misunderstanding that if the self-test were to be performed incorrectly it could give an inaccurate (rather than indeterminate) HIV test result (Supplementary Files, Quote S8).

Another concern about the oral test itself was the potential impact on knowledge of HIV transmission and misinterpretation that saliva could transmit HIV. Several voiced concern that misunderstandings of the mode of transmission would result in greater stigma of MSM, TW, and PLHIV (Supplementary Files, Quote S9).

Finally, there were broader social concerns related to HIVST. This predominantly centred on the concern that employers or other individuals in positions of power would use self-testing for employer-required testing. Similarly to concerns about misunderstanding of saliva-based HIV testing, participants feared that if such compulsory testing were to be applied to key populations, this would result in further stigmatization of MSM, TW, and PLHIV (Supplementary Files, Quote S10).

### Appropriate candidates for HIV self-testing

Given the perceived advantages and disadvantages of HIVST, participants tended to feel that HIVST was potentially more appropriate for some subgroups of MSM and TW in Yangon, according to employment type, age, which was often associated with perceived HIV education and willingness to publicly disclose sexual orientation or gender identity among these groups (Supplementary Files, Quotes S11-13).

Perceptions of appropriate candidates also varied with regard to gender identity and sexual orientation. There was, however, lack of consensus as to whether HIVST would be most appropriate for *Ah Pone* MSM, “closed” MSM who often dress and behave masculine in public and are only known by close friends or family to be MSM, or *Ah Pwint*, “open” MSM who may dress or behave in more feminine manners. This lack of consensus was due to balancing the benefit of privacy with concerns that those with preliminary positive HIVST results would not seek HIV care and treatment nor disclose their status (Supplementary Files, Quotes S14-16).

### Considerations for implementation

In the debate of the merits and drawbacks of HIVST among MSM and TW in Yangon, several suggestions and considerations were provided for future implementation (Table 4). Of most importance was the provision of some form of counselling prior to provision of the HIV self-test kit for the purposes of aiding individual psychosocial response to HIV test results, particularly for those with preliminary positive HIVST results, and providing linkage to confidential, non-stigmatizing treatment and care options for MSM and TW newly identified with HIV infection through HIVST.

**Table 4.** Considerations for implementation of HIV self-testing: key findings and supporting quotations from MSM, TW, and key informants in Yangon, Myanmar

Topic	Relevant quotation
Provision of counseling	*“But there is one thing – some who don’t understand it yet might get wrong because they should get a counselor. If they are going to get the test directly, it should be considered. We have to see whether the saliva testing kits will be handed out only after counseling. If you give out the kits after the counseling, they will have learned about things. And they will know how to use the test kits and what to do when they get the results.” IDI 16, MSM, age 23*
Education about transmission of HIV and how HIVST works	*“You would need to train them before starting this project. You know. You need to put these things into their ears 3 or 4 months in advance. They need to understand in advance that it cannot spread through saliva.” FGD3, Participant 3, MSM community service providers*
Education about transmission of HIV and how HIVST works	*“There is education going on. But discrimination is still there. This device tests saliva. If they don’t have knowledge, there can be more discrimination wondering if it infects from saliva. There should be advertising from TV by Department of Health (giving training) like a short story without giving talks nationwide…. You show them that there are easy methods available. And that although it can be tested from saliva but it cannot be infected from saliva (infection from mosquito bit, sitting, eating pineapple).” FGD5, Participant 4, TW*

Should HIVST be made available to MSM and TW in Myanmar, several participants suggested the use of community education and mass media to inform MSM and TW about appropriate use of HIVST and highlighted the importance of ensuring MSM and TW had access to adequate instructions on how to use the HIV self-test. This education was seen as vital to both ensuring that MSM and TW were well informed but also for mitigating potential misinformation and stigma that could arise from availability of HIVST.

## Discussion

This study aimed to qualitatively assess the acceptability of HIVST among MSM and TW in the greater Yangon Region, Myanmar. HIVST has not yet been approved for over-the-counter use in the country, though the concept of HIVST was met with general acceptability, particularly in regards to the private and confidential nature of the test for a population that has experienced extensive stigma and discrimination. No notable differences were observed between MSM and TW. Rather, differences in perceived benefits and concerns were notable between individuals regardless of gender identity and others tended to perceive that HIVST may be more useful for some subgroups of MSM than others. For MSM, TW, and service providers, the advantages of confidentiality, privacy, individual control of testing, and convenience were, however, weighed against perception that counselling would be absent, potentially impacting mental health and linkage to appropriate confirmatory testing, HIV care and treatment. Additional concerns were further related to fear of misunderstanding that the use of an oral HIV test implies that HIV can indeed be transmitted via saliva.

These merits and concerns related to HIVST are not unique to Myanmar. Other studies have similarly described concerns over the monitoring of self-testing; methods to provide pre- and post-test counselling; access to confirmatory testing and referrals; and potential mental health outcomes of those with a preliminary positive HIVST result [18,30]. Ethical concerns related to potential use for compulsory testing and other harms such as suicide following a preliminary positive HIVST result have also been raised, but have never been borne out in HIVST research [31]. However, in a country with quickly evolving political and cultural environments, concerns and preferences should be considered if/when self-testing is approved in-country and made available in the future. Of salient importance is the provision of some form of HIV counselling, which can be provided with HIVST. To address this, the World Health Organization has proposed two methods for the provision of counseling with HIVST: directly assisted and unassisted HIVST. During directly assisted HIVST, trained providers can provide counselling along with a demonstration of how to perform the test and interpret the result [32]. With unassisted HIVST, the user performs the HIVST on his/her own following the manufacturer provided instructions, which also include instructions for contacting local services for counselling and confirmatory testing [32]. In Myanmar, this requires the availability of well-written Myanmar language instructions that are easy for participants to understand and interpret. Both directly assisted and unassisted methods, however, can incorporate the use of additional methods for counselling and linkage to services, including availability of telephone hotlines, text messages, videos, social media, and other online applications, which have been used for key populations [17,33,34]. Both assisted and unassisted methods are considered screening methods for HIV infection and require confirmatory HIV testing [32]; in Myanmar, HIVST could include direct linkage to NGOs and other MSM and TW friendly HIV services for confirmatory testing and subsequent care.

During the validation workshop, MSM and TW community members further explained the concern that MSM who avoid seeking facility-based HIV testing due to concerns of stigma and discrimination would likely avoid seeking HIV care and treatment following a preliminary positive HIVST results [35]. Thus, to ensure that HIVST is most effective for identifying and engaging MSM and TW in care and regardless of the mechanism in which HIV self-testing is distributed, future implementation should actively develop ways in which to guide participants from HIVST to confirmatory testing and care in a private and confidential manner.

The concern that use of oral HIVST could impact knowledge of HIV transmission and promote HIV stigma suggested that appropriate and accessible health information is an important component of any future scale-up of HIVST in Myanmar. During the validation workshop, MSM and TW community members suggested that participants with lower levels of education encountered greater difficulty in coming to terms with the discrepancy that saliva could be tested for HIV infection but not a means of transmitting the virus [35]. Efforts to prevent misunderstandings may build on suggestions from key informants that other novel forms of community education beyond educational brochures, such as social media and peer networks, be used to educate MSM, TW, and the wider community prior to the implementation of HIVST to proactively address potential misunderstandings and social implications.

Despite concerns, feedback from participants suggested promise for HIVST as an alternative means for early identification of HIV infection among MSM and TW in Myanmar. Given high acceptability of HIVST, including confidentiality, privacy, convenience, comfort, and individual empowerment, such an intervention may improve early diagnosis for a population whose access to HIV services has been hampered by perceived and experienced stigma. Further, the convenience and empowering qualities of HIVST has the potential to facilitate routine HIV testing behaviours, including initiation of HIV testing among naïve testers as well as regular testing for those who are high risk and who have more than 12 months between HIV testing events.

Findings should be reviewed in light of several limitations. This study aimed to qualitatively assess the potential acceptability of HIVST among MSM and TW in Yangon Region, Myanmar. At the time of the study, self-testing was not available in country, thus participant responses are based on their understanding of how HIVST works and perceived advantages and drawbacks. Our subsequent study provides HIVST with pre- and post-test counselling as part of a larger HIV care continuum intervention and will provide further quantitative information on the acceptability and feasibility of use of HIVST. The sample of MSM and TW participants largely comprised individuals who have access to services provided by MSM-friendly NGOs and we do not know how their results would compare to MSM and TW without such access. Our subsequent study will use respondent-driven sampling to identify and recruit participants and may be able to provide further insight into the acceptability among MSM and TW who may not be engaged in MSM and TW friendly NGO services. Finally, conflicts of interest may underlie qualitative research among service providers given their roles in providing HIV testing, counselling, and linkage to care; however, the service providers interviewed for this study come from local community-based non-governmental organizations. These providers are often MSM themselves and have extensive experience serving MSM and TW; thus, their viewpoints related to patterns of HIV testing among MSM and TW and experiences in providing services are important to considering future scale-up of HIV testing approaches for these populations.

## Conclusions

Perceptions about HIVST among MSM, TW, and key informants in Yangon Region, Myanmar highlight considerations related to benefits, drawbacks, and potential implications that must be addressed in future implementation. HIVST provides the confidentiality and privacy that has long been desired for HIV counselling and testing, particularly public facilities; however, HIV counselling and linkage to appropriate services must be well planned to optimize the effectiveness of HIVST for these groups. With appropriate implementation, HIVST could play an important role in reducing undiagnosed infections, improving access to ART, and in improving the overall HIV response for MSM and TW in Myanmar.

## Notes

To access the supplementary material to this article please see Supplementary Files under Article Tools online.
